# A History of Dentistry

**Published:** 1884-10

**Authors:** George H. Perine

**Affiliations:** New York


ARTICLE IV.

### A HISTORY OF DENTISTRY.—THE PROFESSIONAL ADVANCEMENT IN THIS COUNTRY.

BY GEORGE H. PERINE, D. D. S., NEW YORK

The wonderful advancement made in this country in every art and science within that brief space occupying the interval between the opening of the ninteenth century and the present time, has caused the eyes of the old world to turn toward us in astonishment, and the history of this age generations hence will appear to the reader more like an Arabian Nights' tale than of sober truth. Dentistry has more than kept with the other arts and sciences in their advancement toward perfection. The first step in her upward journey at the opening of this century was, we consider, the publication in Baltimore, in 1801, of a treatise on the teeth, by Dr. R. Renner, a practitioner in that city, which is generally believed to have been the first upon the subject published in this country. Following closely upon Renner's little work, came one from the pen of Dr. B. T. Longbotham, also a Baltimore dentist. The object of this work was to enlighten the public regarding the importance of dental treatment, and in it the writer advanced new theories as to the cause of diseases of the teeth and gums. To the life and labors of the distinguished men of which we will speak, the history of our profession in this country is largely indebted. Bringing to its aid minds of extraordinary culture and capability for skill, their influence was deeply felt and gave importance and respect which was widely extended to our then neglected science.

John Randall was born in Massachusetts in 1773. He graduated from Harvard University and commenced practice in Boston about 1805. He was induced to undertake the study of dental surgery during his college career, through, the ignorance displayed regarding that important branch of medical science by a prominent medical man. When Randall began practicing, dental operations consisted almost entirely in the extraction of teeth and the introduction of artificial dentures, little or no attention being paid to the treatment of diseases of the mouth. The number of practitioners who deemed the preservation of the teeth a subject worthy of their consideration was exceedingly limited. Randall, adopting the theory that there is no disease without a remedy, applied himself to the task of discovering a proper mode of treatment for diseases of the gums and carious teeth. By perseverance and study he became proficient in the dental art, which he practiced in connection with the science of medicine. He was particulaaly successful and his active professional labors covered a period of over forty years. He died in 1843.

To give the reader an idea of the crude state of the pro_ fession, or at least the limited knowledge possessed by some of those who undertook its practice, we may call attention to

Leonard Koecker, a German, who began practice in Baltimore in 1807. With a few instruments and an abundance of assurance he made known his intentions. His first operation was the extraction of a tooth which he performed with closed eyes and palpitating heart, very much after the manner of a boy making his first shot with a gun. It is recorded that the patient survived. Koecker, after something of a struggle, met with success, and gained a high reputation as an able and skillful dentist. From necessity, he made many of his instruments and annealed his own gold foil. Leonard Koecker was born at Bremen. in 1785, and while still young he came to this country. In 1822 he returned to Europe and settled in London. Between 1826 and 1835 he published works on Dental Surgery, Artificial Dentures, &c. His death occured in London, August 8th, 1851.

Benjamin James was in successful practice in Boston about this time. He was a man of somewhat exclusive habits. In 1814 he published "A Treatise on the Management of the Teeth."

It was in the year 1816 that John A. Szymanski came to the United States and located in Philadelphia. He afterwards traveled over the states and practiced in many places with success, and did much to advance the specialty in this country. He was born at Warsaw, Poland, in 1793; graduated in medicine at Dantzic, and in 1814 commenced practice. He went to Sweeden and entered the hospital at Gottenburg, where he remained for a time; thence to England, and devoted his attention exclusively to dentistry. Dr. Szymanski possessed a thorough scientific knowledge of his profession, and was a gentlemen of fine education, courtesy and polished bearing. His death occured in January, 1829.

A. A. Plantan came to this country from France, about 1817, and settled in Philadelphia, where he began practice. He was a man of fine education and brilliant attainments. His interest in the advancement of the specialty was marked, and his improvements many. He was the first to introduce and manufacture porcelain teeth, in this country. He was born in 1774, and died in the sixty-fifth year of his age.

About this period, Levi S. Parmly, the oldest of the five brothers, four of whom became dentists, had gained considerable prominence in Boston as a dental practitioner. He was a student under Dr. Petrie, an English dentist, in 1812, and, being a man of superior talents, gained for himself a high professional reputation. In 1815 he went to Montreal, and thence to Quebec, where he practiced for some time; from Canada he returned to the United States, and tarried for a period in the West and South. Later, he made his way to London, where he practiced for a few years. In 1819 he published a work entitled "A Practical Guide to the Management of the Teeth." In 1821 Dr. Parmly delivered lectures in this country on "The Natural History and Management of the Teeth," the causes of their decay, &c., and was also the preceptor of several who afterwards became quite eminent as practitioners, among whom was a younger brother, Dr. Eleazar Parmly. In 1845 Dr. Parmly presented to the profession a valuable paper on Dental Hygiene, which attracted considerable attention. Dr. Parmly returned to Europe, settled in France, and died in Versailles, July 8th, 1859. His remains were brought to this country, and interred at Perry, Ohio, August 29th of the same year, that being the seventieth anniversary of his birthday.

The effort made by active members of the profession to induce the public to realize the importance of dental treatment began at length to produce, in a measure, the desired effect, and practitioners who had hitherto remained inactive awoke to a sense of duty and soon began to assist in every way in their power toward the advancement of professional interests, and "Observations on the Teeth," a small work, was published in 1820 by Dr. Esseveid, of Charleston, S. C., and a second edition of the same work, somewhat enlargeed, appeared a few years later.

In 1824 Dr. Elazar Gedney, who had acquired a high reputation for skillful practice, both in this country and in England, published in Utica, N. Y., where he was then located, a small book bearing the title "The Human Teeth." Gedney's successor in Utica was Dr. A. Blakesley, a skillful dentist, who for years retained the confidence of the community in which he resided.

About this time the number of dental practitioners in the United States did not exceed one hundred, and this number included Dr. John Trenor, of New York, (who, in 1823, contributed to professional literature a paper on the "Teething of Infants," and in 1828 presented his "Physiological Inquiry into the Structure, Organization and Nourishment of the Human Teeth," and a year later his "Observations on Neuralgia" appeared, also "Remarks on Dental Education"); and Dr. Samuel S. Fitch, of New York, (author of "A System of Dental Surgery," a valuable work of reference, published in New York, 1829, the second edition of which was published in Philadelphia, in 1835, whither the writer had removed.)

With each succeeding year the number of the specialty rapidly increased, and with this augmentation naturally came increased knowledge and enlightenment, and a desire on the part of the people for proper dental care so greatly increased the demand for the services of skillful practitioners that many were accustomed to migrate into the rural districts, or make periodical visits to neighboring and in some instances distant cities.

Among these itinerants of worthy professional reputation were Drs. Brewster, Cleveland and Ambler, of South Carolina, Hayden and Harris, of Maryland, Brown and Hale, of Missouri, McReynolds, of Georgia, Badger, of Tennessee, Hullihen, of Virginia, Flagg and Townsend, of Pennsylvania, Keep and Miles, of Massachusetts, Taylor, of Kentucky, Baker and Parmly and Solyman Brown, of New York. The latter, who contributed somewhat liberally to dental literature, will doubtless be remembered by many of the old residents of Long Island, from the fact of his traveling from place to place in considerable state with his chariot and pair, and there is no reason to doubt that a few traces of his skill still remain, reminders of the days when dentistry was little thought of in the rural districts until its missionaries, so to speak, brought it to the very doors of the people, and thus gave them an insight into the benefits it was destined to bestow upon mankind. Not all, however, of those who made professional pastoral pilgrimages left behind them enduring traces of their skill; but often, quite to the contrary, fractured jaws and partly-extracted teeth impressed upon the mind of the unfortunate patient a recollection which years could not remove, and ever after caused the sight of a dentist's *modest sign, in the shape of a somewhat exaggerated gilded tooth*, to produce convulsions of an alarming character. Perhaps the following anecdote, which may be somewhat embellished, but which will give the reader a fair idea of the character and skill of some of the itinerant so-called dentists of the early days of the profession, may prove interesting:

To a certain small hamlet, in the days before railway tracks intersected each other in all parts of the country, like the network of telegraph wires hung in the air like spiders' webs, on a summer morning, there came, with staff in hand and kit on back, a youth who, through the medium of a circular couched in questionable English, announced his willingness to perform for a moderate fee all manner of dental operations, from the extraction of a deciduous incisor to the insertion of artificial dentures likely to cause Dame Nature to blush at her inefficiency in her dental efforts. Several simple operations performed with comparative success were instrumented in procurring for our disciple of Wooffendale a desirable patient in the form of a well-to-do but rather intemperate farmer, whose upper teeth were entirely gone, and who gave our knight of the forceps an order to supply their place with an artificial set, which were accordingly constructed and inserted, and the practitioner, having received his fee, buckled on his kit and set out for the next town. The distance being something like thirty miles, he was obliged to pass a night at a wayside tavern where, the next morning, he was at an early hour called upon by his late patron. "Look a-here," shouted the irate tiller of the soil, confronting the astonished tooth manipulator, "what do yer take me fer, anyhow—a huskin' machine? Look at this 'ere corn-cob what you put inter my mouth yesterday; there ain't a kernel left on the infernel thing," and the indignant rustic produced the bone base from which every tooth had disappeared. "Good heavens!" exclaimed the professional gentlemen in apparent astonishment, and then, as if seized with a sudden thought, he inquired, "What did you eat after those teeth were put into your mouth?"

"Mush and milk," replied the farmer.

"And what did you drink?"

"Apple brandy," was the reply.

"That accounts for it," exclaimed the practitioner, impressively. "You see, sir, the teeth I made you were attached to a base composed of hippopotamus ivory. Now, sir, you must understand that the hippopotamus is an animal which lives a greater part of the time in water. In the temperance sense it is totally an abstemious animal, and when the fiery beverage you are accustomed to came in contact with the fragment of the animal's anatomy to which your artificial teeth were attached, the shock was great, and actually produced so decided a shock among the nerves contained in the bone that the teeth dislodged from their setting."

If not satisfied with our friend's explanation, it is safe to conjecture that the farmer withdrew from the presence of his dentist somewhat mystified.—*New England Journal of Dentistry*.

---

ARTICLE V.

